# The Growing Spectrum of DADA2 Manifestations—Diagnostic and Therapeutic Challenges Revisited

**DOI:** 10.3389/fped.2022.885893

**Published:** 2022-06-14

**Authors:** Carolin Escherich, Benedikt Bötticher, Stefani Harmsen, Marc Hömberg, Jörg Schaper, Myriam Ricarda Lorenz, Klaus Schwarz, Arndt Borkhardt, Prasad Thomas Oommen

**Affiliations:** ^1^Department of Pediatric Oncology, Hematology and Clinical Immunology, University Hospital, Medical Faculty, Heinrich Heine University, Düsseldorf, Germany; ^2^Department of General Pediatrics, Neonatology and Pediatric Cardiology, University Hospital, Medical Faculty, Heinrich Heine University, Düsseldorf, Germany; ^3^Department of Pediatric Hematology and Oncology, Medical Faculty, University of Cologne, Cologne, Germany; ^4^Department of Diagnostic and Interventional Radiology, Medical Faculty, University Hospital, Heinrich Heine University, Düsseldorf, Germany; ^5^Institute for Transfusion Medicine, University Hospital, Medical Faculty, Ulm University, Ulm, Germany; ^6^Institute for Clinical Transfusion Medicine and Immunogenetics Ulm, German Red Cross Blood Service Baden-Württemberg – Hessen, Ulm, Germany

**Keywords:** deficiency of adenosine deaminase type 2, ADA2 enzyme activity, siblings at risk, phenotype-genotype diversity, diagnostic algorithm

## Abstract

Deficiency of Adenosine Deaminase Type 2 (DADA2) is a rare autosomal recessive inherited disorder with a variable phenotype including generalized or cerebral vasculitis and bone marrow failure. It is caused by variations in the adenosine deaminase 2 gene (*ADA2*), which leads to decreased adenosine deaminase 2 enzyme activity. Here we present three instructive scenarios that demonstrate DADA2 spectrum characteristics and provide a clear and thorough diagnostic and therapeutic workflow for effective patient care. Patient 1 illustrates cerebral vasculitis in DADA2. Genetic analysis reveals a compound heterozygosity including the novel *ADA2* variant, p.V325Tfs^*^7. In patient 2, different vasculitis phenotypes of the DADA2 spectrum are presented, all resulting from the homozygous *ADA2* mutation p.Y453C. In this family, the potential risk for siblings is particularly evident. Patient 3 represents pure red cell aplasia with bone marrow failure in DADA2. Here, ultimately, stem cell transplantation is considered the curative treatment option. The diversity of the DADA2 spectrum often delays diagnosis and treatment of this vulnerable patient cohort. We therefore recommend early ADA2 enzyme activity measurement as a screening tool for patients and siblings at risk, and we expect early steroid-based remission induction will help avoid fatal outcomes.

## Introduction

Deficiency of Adenosine Deaminase Type 2 (DADA2) is a rare autosomal recessively inherited disorder resulting in a complex systemic autoinflammatory disease. Its clinical manifestation varies from small- and medium-vessel vasculitis to dysregulation of the immune system and impairment of the hematopoietic system (1–4).

Homozygous or compound heterozygous mutations in the adenosine deaminase 2 gene (*ADA2*) cause a reduction or even absence of adenosine deaminase 2 (ADA2) enzyme activity (3, 4). ADA2 is particularly expressed by myeloid cells and plays a crucial role in the differentiation of monocytes and macrophages (5, 6). Its deficiency further compromises endothelial integrity and development of perivascular inflammation (4, 7). The vasculitis typically manifests in early childhood and presents as recurrent early-onset ischemic stroke or systemic polyarteritis nodosa. Besides mild immunodeficiency, the associated impairment of the immune system may cause various autoinflammatory symptoms. Additionally, the hematopoietic system may be affected, resulting in mild to severe cytopenia of all lineages (anemia, lymphopenia and thrombocytopenia) (1–4, 8–15).

To date, there is no causal therapy for the treatment of ADA2 deficiency. The use of TNF alpha antagonists is currently regarded as first-line treatment (16–18). Encouraging results have been described for patients presenting with a predominant vasculitis phenotype (2, 8, 10, 12, 14, 19, 20). However, the therapeutic effect of TNF alpha antagonizing strategies in patients with severe bone marrow involvement seems to be limited. Cases of successful hematopoietic stem cell transplantation have been described as an alternative definitive treatment option (1, 2, 12, 21).

With regard to the genotype and phenotype of the DADA2 spectrum, more than 100 disease-causing variants have been described so far, leading to a clinical picture ranging from severely affected patients to unaffected carriers who stay asymptomatic into adulthood (2, 11, 13, 16, 22, 23). Here we present three instructive scenarios of ADA2 deficiency that represent the typical clinical patterns: recurrent ischemic strokes (Patient 1), polyarteritis nodosa (Patient 2) and pure red cell aplasia (Patient 3). In addition to the novel *ADA2* variant p.V325Tfs^*^7, described here for the first time, we pay particular attention to a comprehensive diagnostic and therapeutic workflow. This is intended to make early therapy and surveillance strategies available to affected individuals and their at-risk siblings, and to help prevent fatal outcomes.

## Methods

### Patients

We evaluated ADA2 deficiency in three patients and their 1st-degree relatives. All patients and families were followed by the Division of Pediatric Rheumatology at University Hospital Düsseldorf. All patients or their parents provided written informed consent for data collection.

### Genetic Analysis of ADA2 and ADA2 Enzyme Activity

Peripheral blood samples were collected from the index patients and their family members. ADA2 enzyme activity diagnostics were carried out at the Metabolic Laboratory of the Center for Pediatrics and Adolescent Medicine, University Hospital Freiburg, Germany. DNA extraction and ADA2 Sanger sequencing targeted gene analysis was carried out at the Institute for Clinical Transfusion Medicine and Immunogenetics (IKT), Ulm, Germany. ADA2 transcript variants refer to the sequence NM_001282226.1.

### Clinical Assessment

All patients underwent comprehensive clinical and laboratory diagnostics. Immunohistochemical staining was performed on the skin biopsy samples, as well as muscle-nerve biopsies for Patient 2, at the Department of Dermatology and Neuropathology at University Hospital Düsseldorf. Diagnosis of ischemic strokes and polyarteritis nodosa followed standardized diagnostic criteria (24).

## Results

### Patient 1 (Cerebral Vasculitis)

**Figure 1:**
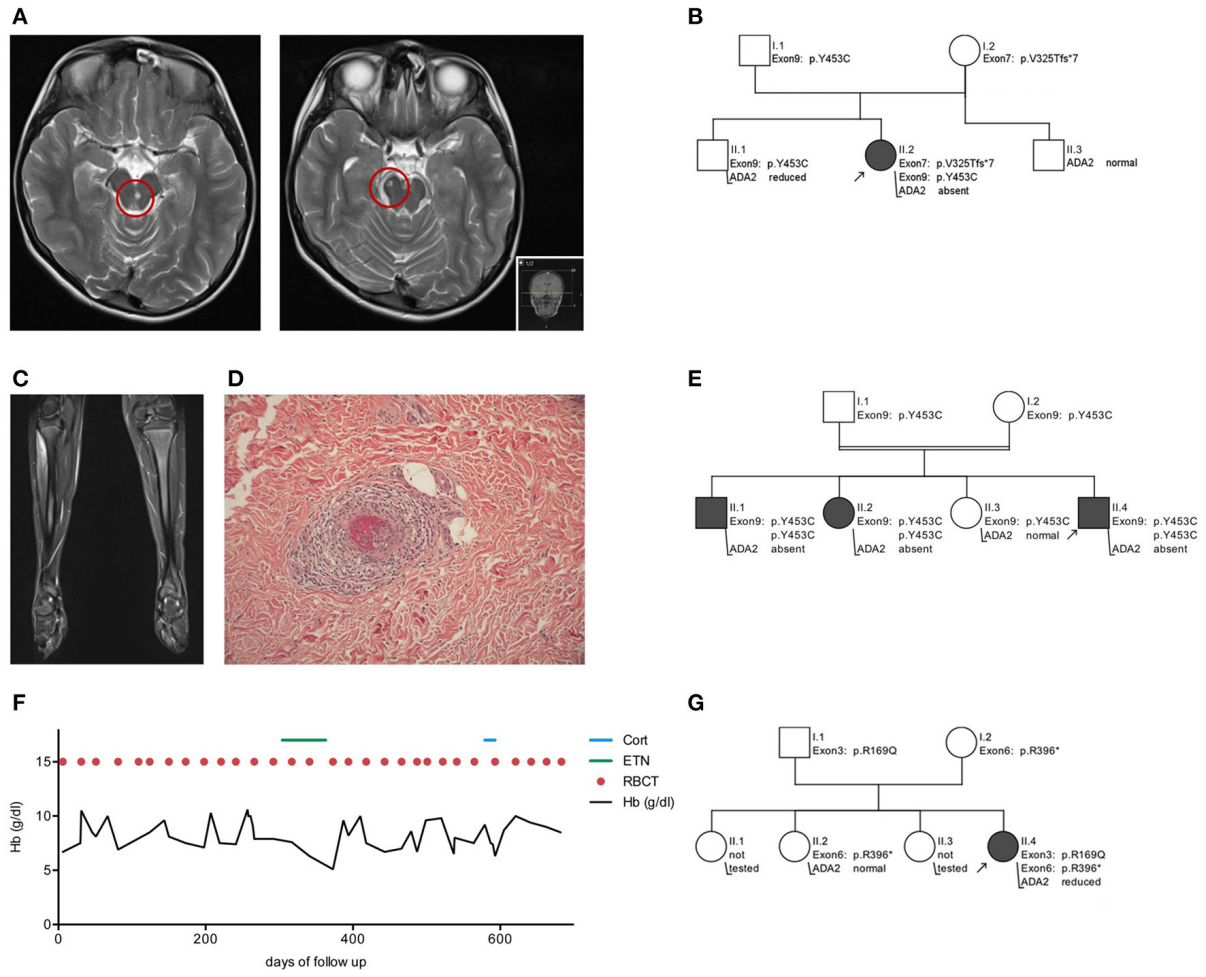
Clinical features and pedigrees of patients with adenosine deaminase 2 deficiency. Patient 1 - Early-onset stroke **(A)** MRI of the brain showing two representative strokes in the mesencephalon right and paramedian left (red circles). **(B)** Illustration of *ADA2* variant inheritance in family 1. Patient 2 - Polyarteriitis nodosa **(C)** MRI of the legs highlights myositis in the right tibialis anterior muscle. **(D)** In skin biopsy polyarteriitis nodosa presents as vasculitis of small cutaneous arteries. **(E)** Illustration of *ADA2* variant inheritance in family 2. Patient 3 - Pure red cell aplasia **(F)** Hb course and transfusion sequence over 2 years of follow up. **(G)** Illustration of *ADA2* variant inheritance in family 3. Hb, hemoglobin concentration; ETN, Etanercept; Cort, cortisol; RBCT, red blood cell transfusion; WT, wild type; Arrow, index patient.

Patient 1, a 10-year-old female of Moroccan descent, initially presented at the age of 9 years with acute double vision and dizziness. Physical examination revealed partial left side oculomotor nerve palsy (CN III) and a disturbance of balance. Cerebral MR imaging confirmed an acute ischemic stroke in the mesencephalon, involving nuclei of the left oculomotor nerve (Figure 1A). Additionally, the patient displayed intermittent internal nuclear ophthalmoplegia, coordination defects and difficulties in concentration and memory. Acute phase reactants were elevated in combination with T-lymphopenia, decreased memory B cells and mild hypogammaglobulinemia (see Table 1). After initiating antithrombotic therapy with acetylsalicylic acid, five additional mesencephalic ischemic strokes occurred within 5 months.

**Table 1:** Characteristics of three patients with adenosine deaminase 2 deficiency.

	**Patient 1**	**Patient 2**	**Patient 3**
Phenotype	Cerebral vasculitis	Childhood polyarteriitis nodosa	Pure red cell aplasia
Genotype *NM_001282226.1*	p.V325Tfs*7 p.Y453C	p.Y453C/p.Y453	p.R169Q p.R306X
ADA2 Activity (mU/mL) *RR 2.7–10.6 mU/mL*	0	0	0.2
CRP (g/dl) *RR <0.5 g/dl*	5	1.5	0.5
SAA (mg/l) *RR <10 mg/l*	338	175	n.d.
ESR (mm/h) *RR <20 mm/h*	26	>140	25
Immunoglobulin Level (mg/dl)			
IgA (RR 52–270 mg/dl)	44	351	200
IgG (RR 670–1,530 mg/dl)	1,030	1,621	690
IgM (RR 62–230 mg/dl)	28	50	40
IgG1 (RR 360–1,120 mg/dl)	902	n.d.	554
IgG2 (RR 89–440 mg/dl)	68	n.d.	41
IgG3 (RR 23–83 mg/dl)	41.2	n.d.	73
IgG4 (RR 5.2–156 mg/dl)	1.2	n.d.	1.4
Immunophenotyping (cell/μl)			
Lymphocytes	1,181	1,645	2,683
T Lymphocytes (CD3+)	909	1,164	1,756
B Lymphocytes (CD20+)	130	197	850
B memory cells (IgD-/CD27+)	1	12.39	1.80

*CRP, C-Reactive Protein; ESR, Erythrocyte sedimentation rate; SAA, Serum Amyloid A; WBC, white blood cell*.

Sanger sequencing of the ADA2 gene revealed a compound heterozygosity (Figure 1B) including exon 7 (p.V325Tfs^*^7, c.973-314_1081+352del) and exon 9 (p.Y453C, c.1358A>G). Notably, the maternally inherited variant p.V325Tfs^*^7 is previously undescribed in the ADA2 gene. Plasma ADA2 enzyme activity was absent (see Table 1). While the absence of ADA2 enzyme activity—as seen in this patient—is typically associated with severe hematological disease, this has also been seen in vasculitis phenotypes (2). Initial treatment comprised a methylprednisolone pulse of 20 mg/kg/d for 5 days, followed by high-dose prednisolone maintenance therapy (2 mg/kg/d). This led to a normalization of the inflammatory parameters. Following weekly treatment with Etanercept s.c. 0.8 mg/kg, the patient has been in continuous clinical remission and steroids have been tapered successfully. During 2 years of follow up, no further ischemic attacks have occurred. Apart from the cerebral manifestation, there was no indication of systemic progression of DADA2.

The pedigree analysis emphasized individually differing ADA2 genotypes and plasma ADA2 activity. The patient's father and brother are both carriers of ADA2 variants. While normal ADA2 activity can be measured in the asymptomatic father, the brother complains of recurrent headache without anomalies in MR imaging. His ADA2 enzyme activity is reduced to 1.78 mU/mL (RR 2.7–10.6 mU/mL). The patient's mother is an asymptomatic carrier of the novel variant p.V325Tfs^*^7 and has decreased ADA2 activity (1.6 mU/mL, RR 2.7–10.6 mU/mL) (Figure 1B).

### Patient 2 (Childhood Polyarteriitis Nodosa)

Patient 2, a 9-year-old male of Moroccan descent, was diagnosed with polyarteritis nodosa (PAN) based on EULAR/PRINTO/PRES c-PAN criteria, including skin involvement, fever, myositis, neuropathy and hypertension at the age of 8 years (24).

He initially presented with a median nerve palsy on the right-hand side and reduced conduction velocity of the right median nerve. Additionally, testing revealed a pathological motor and sensitivity response in the right ulnar nerve, the left sural nerve, the left peroneal nerve and the right tibial nerve, classifying the neurological phenotype as polyneuropathy.

Within 2 months, the symptoms rapidly progressed with decreasing muscle strength. A whole-body MRI revealed myositis in several muscle groups (right forearm, left thigh and lower right leg) (Figure 1C) and a skin biopsy confirmed small vessel vasculitis, causing livedo reticularis and painful subcutaneous nodes (Figure 1D). In line with the morphological pattern of a systemic vasculitis, ultrasound and MR angiographic imaging showed involvement of the coeliac trunk, the superior mesenteric artery and renal arteries on both sides. Acute-phase reactants were elevated, while creatin kinase was within the normal range (26 U/l, RR <248 U/l; see Table 1).

In summary, childhood PAN was diagnosed in this case based on histopathological examination, MR-angiography and clinical findings, using EULAR/PRINTO/PRES c-PAN criteria (24) (Figures 1C,D). Subsequent testing for ADA2 enzyme confirmed its loss of activity, and a homozygous mutation in exon 9 (p.Y453C, c.1358A>G) of ADA2 was found (see Table 1).

As with Patient 1, initial therapy comprised a methylprednisolone pulse on three consecutive days, followed by an oral prednisolone maintenance therapy (1 mg/kg/d). This resulted in a quick and significant improvement of symptoms, leaving only residual sensitivity disorders of the fingers and persistent livedo reticularis of the legs. Finally, treatment with Etanercept s.c. 0.8 mg/kg/weekly led to a complete remission and facilitated steroid tapering. Since initiation of the TNF alpha blockade, no further symptoms have occurred.

**Figure 2:**
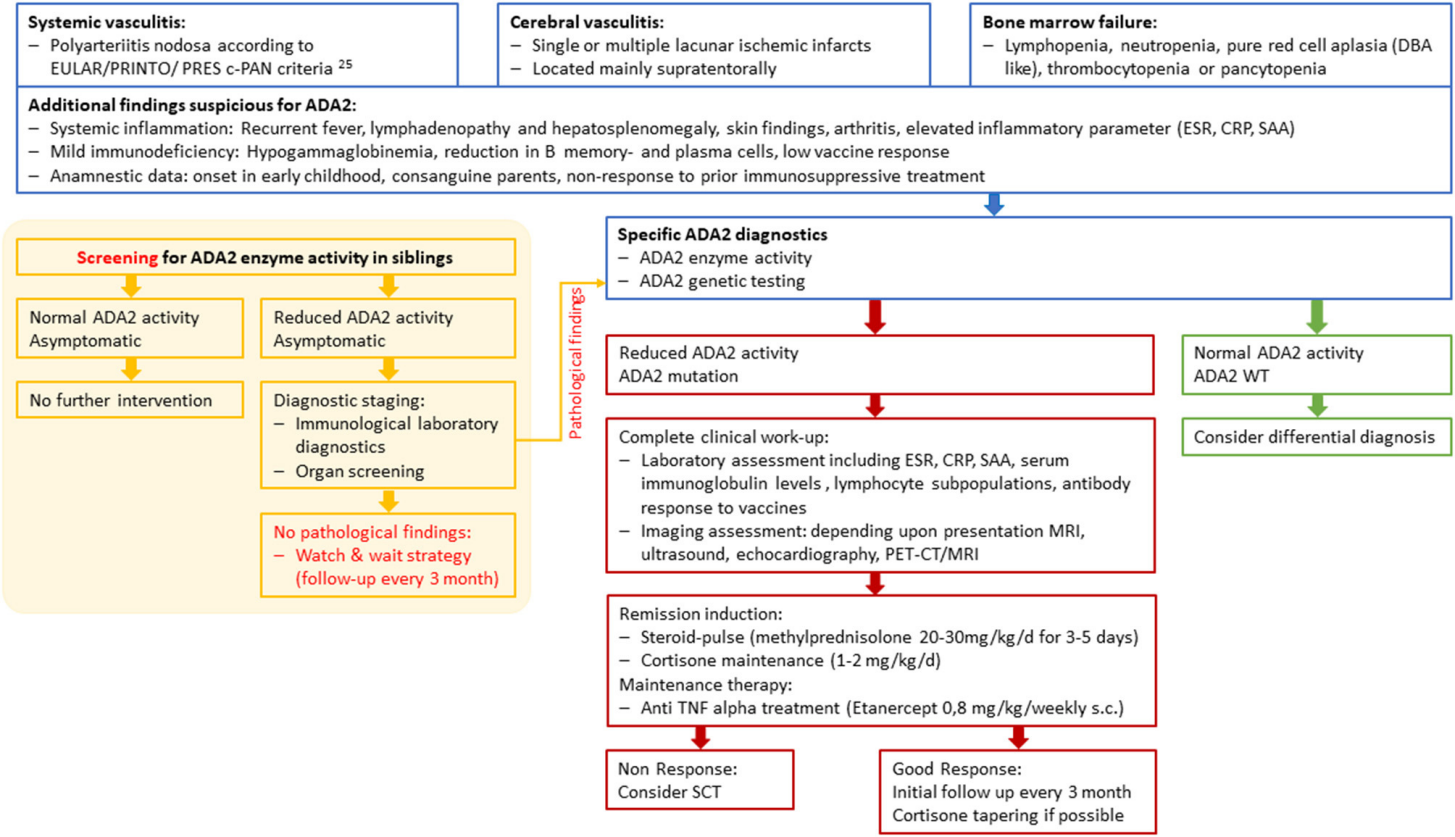
Diagnostic and therapeutic workflow for *ADA2* patients and siblings at risk. c-PAN, childhood polyarteriitis nodosa; DBA, Diamond-Blackfan anemia; ESR, erythrocyte sedimentation rate; CRP, C-Reactive Protein; SAA, Serum Amyloid A; WT, wild type; SCT, Stem cell transplantation.

The pedigree analysis drew attention to the genotype-phenotype correlation within the family. While the index patient developed systemic vasculitis, his older sister suffered from an ischemic stroke at the age of 7 and his older brother complains of sensory paresthesia and suffered from an ischemic stroke at the age of 17. Both are homozygous for the same ADA2 variant, and ADA2 enzyme activity is absent in their plasma (0 mU/ml, RR 2.7–10.6 mU/mL). The consanguine parents and one additional adolescent sister were each tested as asymptomatic heterozygous carriers of the variant p.Y453C (c.1358A>G) (Figure 1E). The sister has not shown any DADA2-related symptoms and has normal ADA2 enzyme activity, although in the low-normal range (3.04 mU/mL; RR 2.7–10.6 mU/mL). Although no further diagnostics or treatments were initiated, the low enzymatic activity means that potential symptoms should be closely monitored. Therefore, the family received intense counseling on recognizing the clinical signs of DADA2 (Figure 2).

### Patient 3 (Pure Red Cell Aplasia)

Patient 3, a 2-year-old girl of Caucasian descent, developed a severe hypo-regenerative anemia during the first weeks of life and continuous dependency on RBC transfusions. After an uneventful pregnancy, the patient was born a mature newborn. She showed age-appropriate development without the appearance of syndromal stigmata. At the age of 3 months, paleness and fatigue appeared. A severe hypo-regenerative normocytic anemia was diagnosed and at hemoglobin levels of 2.6 g/dl, two red blood cell concentrates were administered. The additional blood count was within normal range and no signs of hemolytic anemia were found (LDH, bilirubin, HbF and G6PDH concentrations within normal range).

The patient tested negative for pathogenic variants in genes associated with Diamond-Blackfan anemia: RPS19, RPL5, RPL11, RPS26, RPL35a, and RPL15. Despite initially normal ADA2 activity (which was measured after the initial red blood cell transfusion), a genetic analysis of the ADA2 gene revealed a compound heterozygous mutation involving exon 3 (p.R169Q, c.506G>A) and exon 6 (p.R306^*^, c.916C>T). Repeating the ADA2 enzyme activity analysis revealed a severe reduction to 0.20 mU/mL (see Table 1).

Unremarkable clinical examination, laboratory studies and imaging findings (no fever, no skin involvement, no hepatosplenomegaly, MR angiography without evidence of cerebral vasculopathy) ruled out systemic vasculitis. In accordance with DADA2, further immunological work-up showed a reduced number of memory B cells without clinical signs of pathological susceptibility to infection and immunoglobulin levels within the normal ranges.

After diagnosis, a TNF alpha blocking therapy was initiated with Etanercept s.c. 0.8 mg/kg weekly. No response was seen in the hematological manifestations with a persisting transfusion frequency of every 2–3 weeks (Figure 1F). After 6 months, TNF alpha blocking therapy was discontinued. The subsequent steroid treatment did not improve the transfusion frequency either, and therefore stem cell transplantation is currently being considered as a curative treatment option.

Through genetic analysis, the patient's parents and one sister were found to be asymptomatic heterozygous carriers of an ADA2 variant in either exon 3 or exon 6 (Figure 1G).

## Discussion

DADA2 was first described in 2014 as a rare early onset autoinflammatory disease (3, 4). Since then, over 300 cases of DADA2 have been published and, over time, a wide variety of symptoms and pathogenic genetic variants have become visible (2, 8, 16, 17, 22, 23). In this compilation, we present three families with different ADA2 variants leading to the typical manifestations of the DADA2 spectrum. We identify a novel ADA2 variant, present a diagnostic and therapeutic workflow to improve rapid treatment initiation and address the therapeutic dilemma in homozygous but seemingly unaffected siblings.

To date, more than 100 variants have been described in the ADA2 gene (2, 22). With p.Y453C (Patients 1 & 2) and p.R169Q (Patient 3), we present two disease-causing missense mutations that are among the most common ADA2 variants described so far (2, 17). Additionally, in Patient 1 we present a compound heterozygous inheritance pattern including a novel ADA2 variant (Figure 1B). This 773 bp genomic deletion includes the entire exon 7 and leads to a frameshift mutation with a premature stop codon. Several variants with Exon 7 deletion have been described in the context of ADA2 deficiency (2). Interestingly, a comparable genetic constellation has been previously reported in two siblings with similar phenotype (25).

In Patient 1, we also find an intron variant on the paternal allele. While intron mutations have previously been assigned to splice variants of ADA2 and might affect protein expression and enzyme activity, to date we do not know the consequences of this variant (9, 12, 13).

The DADA2 phenotype can a priori be divided into three clinical manifestation entities: polyarteritis nodosa, ischemic stroke and bone marrow failure. These may be accompanied by a mild immunodeficiency with reduction of memory B cells, plasma cells and hypogammaglobulinemia (3–5, 16–18, 22, 23). Previous studies aimed to establish a correlation between genotype and phenotype. For example, Ozen et al. reported a correlation between ADA2 variants in the catalytic domain and the DBA phenotype, whereas in PAN-like manifestation, the dimerization domain might be affected (12). This observation has not been confirmed by larger studies. A systematic analysis of over 150 cases encompassing all groups of the DADA2 spectrum revealed that ADA2 variants of each phenotype were scattered throughout the entire gene. However, missense variants with residual ADA2 activity are commonly seen in vasculitis phenotypes, whereas bone marrow failure is associated with missense, non-sense or frameshift variants and complete loss of ADA2 activity (2). In family 2, we present three siblings with identical genotypes but different phenotypes. As a result, their manifestations appeared at different ages, at different intensities and with a different spectrum of symptoms. Therefore, our findings are in line with larger studies, which support the hypothesis that diversity of DADA2 phenotypes cannot be explained by the genotype alone, but that further genetic or epigenetic modifiers are likely to affect DADA2 expressivity (2, 11, 12, 17, 26).

Immunosuppressive agents are commonly used to control systemic inflammation. Steroids lead to a short-term response, but with flares of inflammation upon tapering (3, 4, 10). For long-term treatment, several classic immunosuppressive drugs have been insufficient in controlling disease activity (8, 10). ADA2 is produced and secreted by activated monocytes, macrophages, and dendritic cells, performing an important function in immune regulation (5, 6). ADA2 deficiency results in a predominance of M1 macrophages characterized by increased production of pro-inflammatory cytokines, such as tumor necrosis factor alpha (TNF alpha), an important mediator of vasculitis and tissue damage (4, 5, 8, 27, 28). Anti-TNF alpha therapy is well-established in the treatment of juvenile idiopathic arthritis and inflammatory bowel disease (29–31). In DADA2 treatment, anti-TNF alpha agents lead to an improvement of the inflammatory vasculitis phenotype (5, 8, 12, 19, 28). In accordance with current and published experiences in DADA2-treatment (16, 19, 22, 23), we initiated anti-TNF alpha treatment in all three patients as soon as DADA2 was diagnosed. The vasculitis phenotypes in Patients 1 and 2 showed good responses and no further complications occurred. By contrast, neither anti-TNF therapy nor steroids showed any effect on the hematological phenotype in Patient 3. In such cases, stem cell transplantation is currently the only alternative treatment option, leading to resolution of the hematological and immunological phenotype and normalization of ADA2 plasma enzyme activity (1, 2, 12, 22, 23). Considering the serious potential side effects, this therapeutic alternative should be discussed very thoroughly and should only be considered in cases of non-response to first-line anti-TNF alpha treatment (1, 17, 18).

So far, there are no guidelines clarifying the indication for anti-TNF alpha therapy. Some authors recommend treatment initiation when biallelic ADA2 variants coincide with the absence of catalytic ADA2 activity in both symptomatic and asymptomatic patients, to reduce the risk of ischemic stroke and prevent severe neurological complications (8, 11, 14, 16, 20). On the other hand, anti-TNF alpha treatment may also be accompanied by serious side effects, such as infection, induction of autoinflammation and a potentially higher risk of malignancies (29, 31). Altogether, we advocate for the early use of TNF inhibition in a (cerebral) vasculitis DADA2-phenotype, facilitating steroid-free long-term clinical care (Figure 2). Elevated acute-phase reactants may serve as surrogate parameters in order to monitor treatment response.

Early diagnosis is often hampered by the diverse and non-specific symptoms associated with the DADA2 spectrum. This is particularly seen in Patient 1, who developed five ischemic strokes within 5 months under anticoagulation treatment. Our recommendation for immediate diagnostic testing when there is clinical evidence of DADA2 includes the measurement of ADA2 enzyme activity. In addition, genetic testing should be carried out to confirm a biallelic loss-of-function mutation in the ADA2 gene (Figure 2). ADA2 enzyme activity measurement may also offer a useful tool for screening for DADA2 in patients with a suspicious clinical history or in unaffected siblings prior to genetic testing (22). The question of how to proceed with low or absent ADA2-activity in seemingly unaffected siblings currently remains unresolved. We strongly suggest a thorough diagnostic work-up in these individuals, including immunological laboratory investigations, as well as organ screening, including CNS, heart diagnostics and assessment of clinical signs of c-PAN (Figure 2). As the use of anticoagulants may increase the chances of an ischemic stroke turning into a hemorrhagic stroke, these substances have to be applied rather cautiously (32).

In summary, this overview on DADA2 signs and symptoms outlines the main characteristics and current challenges of the DADA2 disease spectrum. The establishment of standardized guidelines would further improve early diagnosis and treatment regimens, while also preventing severe complications. We consider early measurement of ADA2 activity and thorough organ screening of patients and seemingly unaffected family members to be indispensable components of the diagnostic process.

## Data Availability Statement

The original contributions presented in the study are included in the article/supplementary material, further inquiries can be directed to the corresponding author/s.

## Ethics Statement

Written informed consent was obtained from the minor(s)' legal guardian/next of kin for the publication of any potentially identifiable images or data included in this article.

## Author Contributions

Data collection and analysis were performed by CE and PTO. The first draft of the manuscript was written by CE and all authors commented on previous versions of the manuscript. All authors contributed to the study conception and design. All authors read and approved the final manuscript.

## Conflict of Interest

The authors declare that the research was conducted in the absence of any commercial or financial relationships that could be construed as a potential conflict of interest.

## Publisher's Note

All claims expressed in this article are solely those of the authors and do not necessarily represent those of their affiliated organizations, or those of the publisher, the editors and the reviewers. Any product that may be evaluated in this article, or claim that may be made by its manufacturer, is not guaranteed or endorsed by the publisher.
